# Supplementary material for: Unraveling the thermodynamic criteria for size-dependent spontaneous phase separation in soft porous crystals
Source: Nat Commun. 2019 Oct 24;10:4842. doi: 10.1038/s41467-019-12754-w (PMC6813293; doi:10.1038/s41467-019-12754-w)
Supplement: Supplementary file 1 — Supplementary Information [file 41467_2019_12754_MOESM1_ESM.pdf]

Supplementary Information for

Unraveling the Thermodynamic Criteria for Size-Dependent Spontaneous Phase Separation in Soft Porous Crystals

Rogge et al.

Supplementary Note 1 – Thermodynamic model for pressure and free energy equations of state of SPCs containing phase coexistence regions 3

Supplementary Note 2 – Size comparison between theoretical and experimental mesocells 8

Supplementary Note 3 – Definition of the subcells within a given simulation cell 9

Supplementary Note 4 – Correlation between neighboring subcells in MIL-53(Al) 11

Supplementary Note 5 – Effect of the cell size on the MIL-53(Al) transition pressures 14

Supplementary Note 6 – Constructing the free enthalpy equations of state 17

Supplementary Note 7 – Convergence of the obtained equations of state 20

Supplementary Note 8 – Robustness analysis of the obtained equations of state 25

Supplementary Note 9 – Simulated X-ray diffraction patterns for the phase coexistence regions 29

Supplementary Note 1 – Thermodynamic model for pressure and free energy equations of state of SPCs containing phase coexistence regions

Assume a soft porous crystal (SPC) exhibiting exactly two (meta)stable phases at a given temperature and at a pressure of 0 MPa. Furthermore, assume that the SPC consists of n_{layer} layers, of which n_{cp} layers are in the closed-pore phase and $n_{\text{lp}} = n_{\text{layer}} - n_{\text{cp}}$ layers are in the large-pore phase. Let K_{cp} and K_{lp} be the bulk moduli of the two phases, and V_{cp} and V_{lp} be their equilibrium volumes. Furthermore, introduce

$$x_{\text{cp}} = \frac{n_{\text{cp}}}{n_{\text{layer}}} \qquad \text{and} \qquad x_{\text{lp}} = \frac{n_{\text{lp}}}{n_{\text{layer}}}, \tag{1}$$

such that $x_{\text{cp}} + x_{\text{lp}} = 1$ and $0 \leq x_{\text{cp}}, x_{\text{lp}} \leq 1$. In this supplementary note, a simple thermodynamic model will be established to predict the pressure and free energy equations of state of this SPC given the possibility of phase coexistence, only using information on the two pure-phase equilibria (equilibrium volumes, bulk moduli and their derivatives, and the free energy difference between the cp and lp phases) and the free energy barrier associated with creating an lp/cp interface. This model allows one to extract the most important factors driving phase coexistence in SPCs, and can be adopted to predict phase coexistence in crystals too large to be directly simulated, even when using force fields.

Obtaining the volume distribution for a phase coexistence region

Given that the cp and lp phases may coexist in the SPC, a given volume V of the conventional unit cell can be obtained through various combinations of volumes V_1 of the cp phase (close to the cp equilibrium volume V_{cp}) and volumes V_2 of the lp phase (close to the lp equilibrium volume V_{lp}) , as long as these volumes satisfy

$$V = x_{\text{cp}} V_1 + x_{\text{lp}} V_2. \tag{2}$$

The free energy for this material composed of the two phases can then be expressed as

$$F(x_{\text{cp}}, x_{\text{lp}}; V_1, V_2) = x_{\text{cp}} \frac{K_{\text{cp}}}{2V_{\text{cp}}} (V_1 - V_{\text{cp}})^2 + x_{\text{lp}} \frac{K_{\text{lp}}}{2V_{\text{lp}}} (V_2 - V_{\text{lp}})^2. \tag{3}$$

Through substitution of Supplementary Equation 2, the variable V_2 can be replaced in the above

equation, yielding

$$F(x_{\rm cp}, x_{\rm lp}; V, V_1) = x_{\rm cp}\frac{K_{\rm cp}}{2V_{\rm cp}}(V_1 - V_{\rm cp})^2 + x_{\rm lp}\frac{K_{\rm lp}}{2V_{\rm lp}}\left(\frac{V - x_{\rm cp}V_1}{x_{\rm lp}} - V_{\rm lp}\right)^2. \tag{4}$$

In this derivation, we assumed $x_{\rm lp} \neq 0$; for $x_{\rm lp} = 0$ only the cp phase exists and the construction of the free energy expression is trivial.

To find the composition (V_1, V_2) for which the free energy F is minimized at a given average volume V, we take the derivative of Supplementary Equation 4 with respect to V_1:

$$\frac{\partial F(x_{\rm cp}, x_{\rm lp}; V, V_1)}{\partial V_1} = x_{\rm cp}\frac{K_{\rm cp}}{V_{\rm cp}}(V_1 - V_{\rm cp}) - x_{\rm cp}\frac{K_{\rm lp}}{V_{\rm lp}}\left(\frac{V - x_{\rm cp}V_1}{x_{\rm lp}} - V_{\rm lp}\right). \tag{5}$$

Requiring this derivative to equal zero yields

$$x_{\rm lp}V_{\rm lp}K_{\rm cp}(V_1 - V_{\rm cp}) = V_{\rm cp}K_{\rm lp}(V - x_{\rm cp}V_1 - x_{\rm lp}V_{\rm lp}), \tag{6}$$

or, solved for V_1:

$$V_1(x_{\rm cp}, x_{\rm lp}; V) = \frac{K_{\rm lp}V_{\rm cp}V + x_{\rm lp}(K_{\rm cp} - K_{\rm lp})V_{\rm lp}V_{\rm cp}}{x_{\rm lp}K_{\rm cp}V_{\rm lp} + x_{\rm cp}K_{\rm lp}V_{\rm cp}}. \tag{7}$$

This expression reveals that, if the cp phase were to be extremely stiff, i.e., $K_{\rm cp} \gg K_{\rm lp}$, then $V_1 \approx V_{\rm cp}$: the variation in V only stems from the variation V_2 of the softer phase.

Free energy equation of state for a phase coexistence region

Inserting the composition that minimizes the free energy at a given volume V from Supplementary Equation 7 into Supplementary Equation 4, we obtain a first estimate of the free energy of the material with fixed composition $(n_{\rm cp}, n_{\rm lp})$:

$$\begin{aligned} F(x_{\rm cp}, x_{\rm lp}; V) &= x_{\rm cp}\frac{K_{\rm cp}}{2V_{\rm cp}}(V_1(V) - V_{\rm cp})^2 + \frac{1}{x_{\rm lp}}\frac{K_{\rm lp}}{2V_{\rm lp}}\left(V - x_{\rm cp}V_1(V) - x_{\rm lp}V_{\rm lp}\right)^2 && (8)\\ &= x_{\rm cp}\frac{K_{\rm cp}K_{\rm lp}^2V_{\rm cp}}{2(x_{\rm lp}K_{\rm cp}V_{\rm lp} + x_{\rm cp}K_{\rm lp}V_{\rm cp})^2}(V - x_{\rm lp}V_{\rm lp} - x_{\rm cp}V_{\rm cp})^2 \\ &\quad + x_{\rm lp}\frac{K_{\rm lp}K_{\rm cp}^2V_{\rm lp}}{2(x_{\rm lp}K_{\rm cp}V_{\rm lp} + x_{\rm cp}K_{\rm lp}V_{\rm cp})^2}\left(V - x_{\rm cp}V_{\rm cp} - x_{\rm lp}V_{\rm lp}\right)^2 && (9)\\ &= \frac{K_{\rm lp}K_{\rm cp}}{2(x_{\rm lp}K_{\rm cp}V_{\rm lp} + x_{\rm cp}K_{\rm lp}V_{\rm cp})}\left(V - x_{\rm cp}V_{\rm cp} - x_{\rm lp}V_{\rm lp}\right)^2. && (10) \end{aligned}$$

Note that, in the limiting case $x_{\mathrm{cp}} = 0$, $x_{\mathrm{lp}} = 1$ and the above equation simplifies to

$$F(x_{\mathrm{cp}} = 0, x_{\mathrm{lp}} = 1; V) = \frac{K_{\mathrm{lp}}}{2V_{\mathrm{lp}}} \left(V - V_{\mathrm{lp}}\right)^2, \tag{11}$$

retrieving the ideal free energy equation of state for a pure lp material. A similar expression can be found for $x_{\mathrm{lp}} = 0$. As indicated below, a correction term is added to the equation of state (Supplementary Equation 10) for the pure cp and lp phases.

In the above expression, all coexistence regions would attain the same free energy minimum, in contradiction with experimental observations and the modelled free energy profile in which a free energy barrier $\Delta F_{\mathrm{lp-cp}}$ between the lp and cp phase is present (according to the used force field, $\Delta F_{\mathrm{lp-cp}} = 24.5$ kJ mol^{-1} for MIL-53(Al) at 300 K). To introduce this free energy barrier in a continuous fashion over all coexistence regions, a term is added to Supplementary Equation 10, yielding the following equation of state for the phase coexistence regions:

$$F_{\mathrm{int}}(x_{\mathrm{cp}}, x_{\mathrm{lp}}; V) = x_{\mathrm{lp}} \Delta F_{\mathrm{lp-cp}} + \frac{K_{\mathrm{lp}} K_{\mathrm{cp}}}{2(x_{\mathrm{lp}} K_{\mathrm{cp}} V_{\mathrm{lp}} + x_{\mathrm{cp}} K_{\mathrm{lp}} V_{\mathrm{cp}})} \left(V - x_{\mathrm{cp}} V_{\mathrm{cp}} - x_{\mathrm{lp}} V_{\mathrm{lp}}\right)^2. \tag{12}$$

Pressure equation of state for a phase coexistence region

The pressure equation of state for each of the $(n_{\mathrm{cp}}, n_{\mathrm{lp}})$ phase coexistence regions can readily be obtained from Supplementary Equation 12 as:

$$P_{\mathrm{int}}(x_{\mathrm{cp}}, x_{\mathrm{lp}}; V) = -\frac{\partial F_{\mathrm{int}}(x_{\mathrm{cp}}, x_{\mathrm{lp}}; V)}{\partial V} \tag{13}$$

$$= -\frac{K_{\mathrm{lp}} K_{\mathrm{cp}}}{x_{\mathrm{lp}} K_{\mathrm{cp}} V_{\mathrm{lp}} + x_{\mathrm{cp}} K_{\mathrm{lp}} V_{\mathrm{cp}}} \left(V - x_{\mathrm{cp}} V_{\mathrm{cp}} - x_{\mathrm{lp}} V_{\mathrm{lp}}\right). \tag{14}$$

For the intermediate phase coexistence regions, this linear expression as a function of the volume is provided as grey dotted lines in Supplementary Figure 1. To reproduce well the transition pressures of the pure lp and cp phases and capture the stronger curvature of the pressure equation of state in these regions, however, a higher-order approximation is necessary. To this end, the second- and third-order derivatives of the pressure with respect to the volume are determined at the cp and lp equilibrium volumes. These higher-order derivatives are employed to further refine Supplementary Equation 14 for the pure-phase regions:

$$P_{\mathrm{cp}}(V) = P_{\mathrm{int}}(x_{\mathrm{cp}} = 1, x_{\mathrm{lp}} = 0; V) + \frac{1}{2} \left.\frac{\partial^2 P}{\partial V^2}\right|_{V=V_{\mathrm{cp}}} (V - V_{\mathrm{cp}})^2 + \frac{1}{6} \left.\frac{\partial^3 P}{\partial V^3}\right|_{V=V_{\mathrm{cp}}} (V - V_{\mathrm{cp}})^3, \tag{15}$$

and

$$P_{\mathrm{lp}}(V) = P_{\mathrm{int}}(x_{\mathrm{cp}} = 0, x_{\mathrm{lp}} = 1; V) + \frac{1}{2} \left.\frac{\partial^2 P}{\partial V^2}\right|_{V=V_{\mathrm{lp}}} (V - V_{\mathrm{lp}})^2 + \frac{1}{6} \left.\frac{\partial^3 P}{\partial V^3}\right|_{V=V_{\mathrm{lp}}} (V - V_{\mathrm{lp}})^3. \quad (16)$$

The corresponding free energy profiles for the cp and lp phases are then given by:

$$F_{\mathrm{cp}}(V) = F_{\mathrm{int}}(x_{\mathrm{cp}} = 1, x_{\mathrm{lp}} = 0; V) - \frac{1}{6} \left.\frac{\partial^2 P}{\partial V^2}\right|_{V=V_{\mathrm{cp}}} (V - V_{\mathrm{cp}})^3 - \frac{1}{24} \left.\frac{\partial^3 P}{\partial V^3}\right|_{V=V_{\mathrm{cp}}} (V - V_{\mathrm{cp}})^4, \quad (17)$$

and

$$F_{\mathrm{lp}}(V) = F_{\mathrm{int}}(x_{\mathrm{cp}} = 0, x_{\mathrm{lp}} = 1; V) - \frac{1}{6} \left.\frac{\partial^2 P}{\partial V^2}\right|_{V=V_{\mathrm{lp}}} (V - V_{\mathrm{lp}})^3 - \frac{1}{24} \left.\frac{\partial^3 P}{\partial V^3}\right|_{V=V_{\mathrm{lp}}} (V - V_{\mathrm{lp}})^4. \quad (18)$$

Inserting the interfacial free energy barrier due to phase coexistence

Until now, it has been assumed that forming an lp/cp interface does not introduce an interfacial strain and an associated free energy penalty. However, this is in disagreement with the local deformations at the inorganic nodes observed at the lp/cp interfaces and would lead our model to overstabilize the phase coexistence regions with respect to the force field results. Based on the force field results for the different coexistence regions in the 4×2×4, 6×2×6, and 8×2×8 supercells for MIL-53(Al), an interfacial free energy barrier amounting to

$$\Delta F_{\mathrm{interface}} = \frac{11.25 \text{ kJ mol}^{-1}}{\sqrt{n_{\mathrm{layer}}}} \quad (19)$$

per interface is introduced. While our periodic boundary conditions always impose an even number of interfaces, the thermodynamic model can also consider an isolated crystal, for which an odd number of interfaces can be present. The interfacial free energy barrier decreases with the number of layers, as the relative importance of the interfacial strain decreases the larger the crystal size. Finally, given that the strain fields of neighboring interfaces may partially cancel if they are sufficiently close together, the interfacial free energy barrier is reduced to 50%, 80%, or 90% of its original value if the two interfaces are separated by one, two, or three intermediate layers.

Obtaining the overall free energy and pressure equations of state

The above thermodynamic model provides the pressure and free energy of each separate phase coexistence region of an SPC. These intermediate results are indicated with dotted grey lines in Supplementary Figure 1, showing already good piecewise agreement with overall pressure and free energy equations of state. To obtain from these partial equations of state an overall and final

free energy and pressure equation of state, it is assumed that two neighboring phase coexistence regions may dynamically coexist over time. In that case, we can interpolate between neighboring phases, as commonly done for phase mixtures. This interpolation yields the final free energy and pressure equations of state predicted by the thermodynamic model, in good agreement with the profiles obtained based on atomistic modelling.

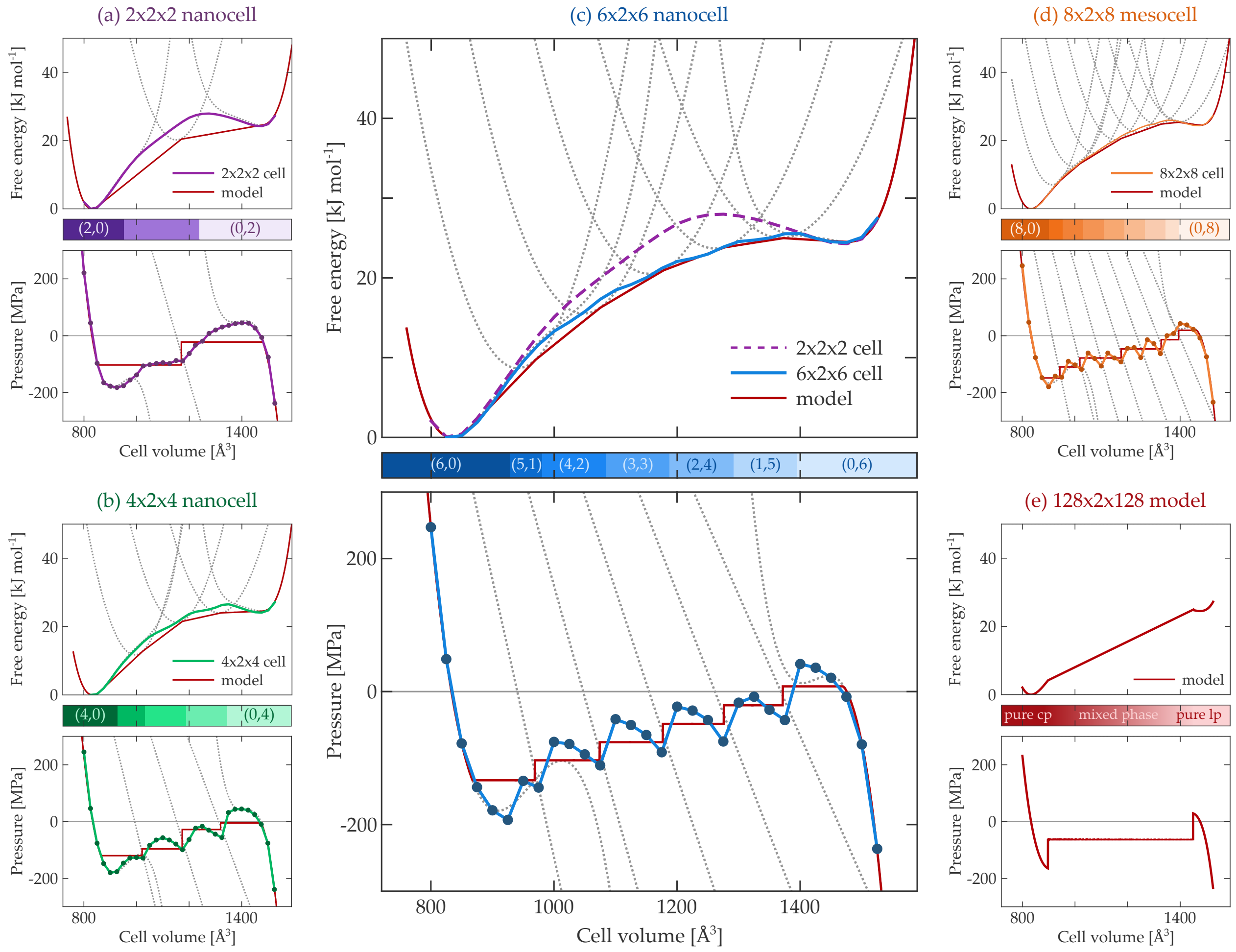

Supplementary Figure 1: Pressure (bottom) and free energy (top) equations of state, with indication of the metastable (n_{cp},n_{lp}) phase coexistence regions, in which n_{cp} cp and n_{lp} lp layers coexist, as a function of the volume (middle) for four different cell sizes: (a) a 2×2×2 supercell, (b) a 4×2×4 supercell, (c) a 6×2×6 supercell, and (d) an 8×2×8 supercell, all at 300 K (see also Figure 3 of the main text). The red lines indicate the fitted thermodynamic model, which is extrapolated in (e) for a 128×2×128 cell (208.0 × 1.4 × 171.2 nm^3), similar in size to experimental MIL-53(Al)-NH_2 crystals. The grey dotted lines correspond to intermediate results obtained by the thermodynamic model for each phase coexistence region separately.

Supplementary Note 2 – Size comparison between theoretical and experimental mesocells

Supplementary Table 1 provides an overview of the different cell sizes for the materials discussed in the manuscript. All cell sizes correspond to the lp phase, which were used to initiate the initial $(N, P, \boldsymbol{\sigma}_\mathrm{a} = \mathbf{0}, T)$ simulations. Furthermore, the last lines of Supplementary Table 1 list the sizes of experimental mesosized soft porous crystals. Herein and in those references, the term 'mesocell' has been used consistently for crystals with a cell size between 10 nm and 1 μm. For smaller crystals, that are traditionally used in simulations, we adopted the term nanocells.

Supplementary Table 1: Size of the simulation cells considered in this manuscript and corresponding mesocell sizes of experimentally synthesized SPC crystals. †Not a single crystal. ‡SPC without the pillared-layered structure. BDC = benzene-1,4-dicarboxylate, BDP = benzene-1,4-dipyrazolate, BME-BDC = 2,5-bis(2-methoxyethoxy)benzene-1,4-dicarboxylate, BPE = 1,2-bis(4-pyridyl)ethylene, BPY = 4,4'-bipyridine, DABCO = 1,4-diazabicyclo[2.2.2]octane, DE-BDC = 2,5-diethoxybenzene-1,4-dicarboxylate, DUT = Dresden University of Technology, MIL = Matériaux de l'Institut Lavoisier.

Material	Source	Size	Nomenclature
MIL-53(Al) (1×2×1)	This work (theoretical)	1.6×1.4×1.3 nm^3	nanocell
MIL-53(Al) (2×2×2)	This work (theoretical)	3.2×1.4×2.7 nm^3	nanocell
MIL-53(Al) (4×2×4)	This work (theoretical)	6.5×1.4×5.4 nm^3	nanocell
MIL-53(Al) (6×2×6)	This work (theoretical)	9.7×1.4×8.0 nm^3	nanocell
MIL-53(Al) (8×2×8)	This work (theoretical)	13.0×1.4×10.7 nm^3	mesocell
DMOF-1(Zn) (8×2×8)	This work (theoretical)	12.6×1.9×12.6 nm^3	mesocell
MIL-53(Al)-F (8×2×8)	This work (theoretical)	13.2×1.3×10.6 nm^3	mesocell
CoBDP (8×2×8)	This work (theoretical)	15.1×1.4×15.1 nm^3	mesocell
Cu_2(DE-BDC)$_2$DABCO†	Ref. 1 (experimental)	$\varnothing = 37$ nm	mesocell
[Cu_2(BDC)$_2$(BPY)]$_n$	Ref. 2 (experimental)	50×20×50 nm^3	mesocell
DUT-49(Cu)‡	Ref. 3 (experimental)	$\varnothing = 107$ nm	mesocell
MIL-53(Al)-NH_2	Ref. 4 (experimental)	210×100×210 nm^3	mesocell
DUT-8(Ni)	Ref. 5 (experimental)	$\varnothing = 500$ nm	mesocell
[Cu_2(BDC)$_2$(BPE)]$_n$	Ref. 2 (experimental)	700×80×700 nm^3	mesocell

Supplementary Note 3 – Definition of the subcells within a given simulation cell

To visualize the different regions in Figure 2 and the Supplementary Movies, a volume has been assigned to each 1×1×1 subcell of the different simulation cells. These subcells were constructed by randomly selecting a metal atom – aluminum for MIL-53(Al) and MIL-53(Al)-F, zinc for DMOF-1(Zn), and cobalt for CoBDP – and identifying the metal atoms that would be equivalent when considering a 1×1×1 unit cell with periodic boundary conditions. As all $(N, P, \boldsymbol{\sigma}_a = \mathbf{0}, T)$ simulations started from a perfectly ordered structure obtained by periodically repeating a 1×1×1 unit cell, this assignation is unique.

From this set of metal atoms, a subcell is defined by considering a given metal atom and its neighbors in the positive x, y, and z directions (see Supplementary Figure 2). Based on these four atoms, three subcell vectors are defined and collected into the subcell matrix. Finally, the volume of this subcell is calculated as the determinant of the subcell matrix. This procedure is repeated for each of the subcells present in the considered simulation cell. This procedure is visualized for a 2D system consisting of two subcells in Supplementary Figure 2. The orange subcell is defined by the vectors between atoms 2 and 1 on the one hand and between atoms 4 and 1 on the other hand. Similarly, the blue subcell is defined by the vectors between atoms 3 and 2 on the one hand and between atoms 5 and 2 on the other hand.

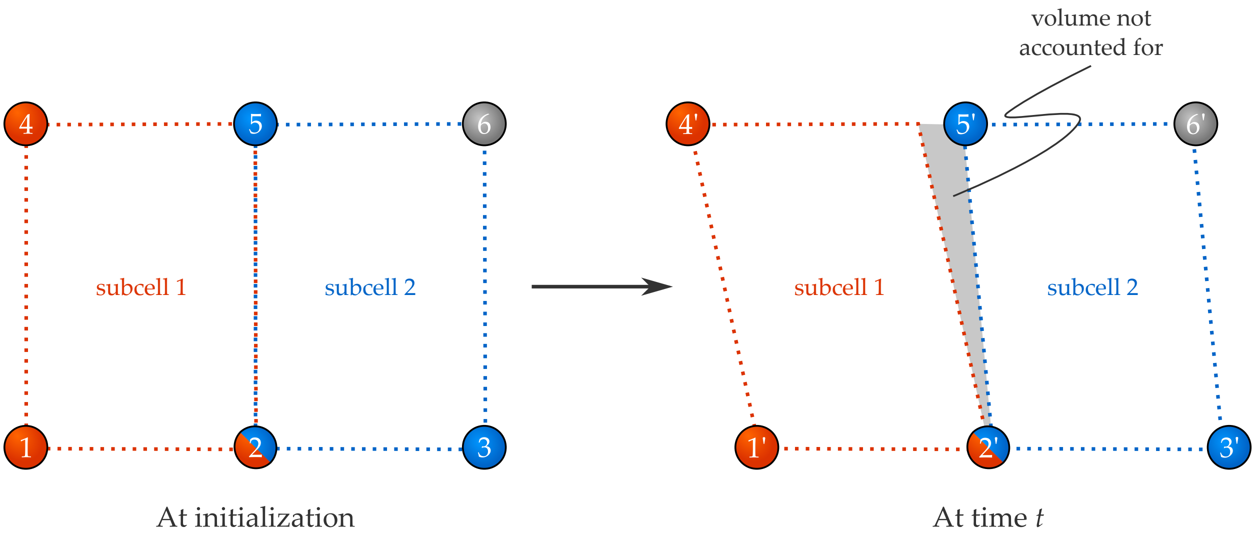

Supplementary Figure 2: Schematic 2D overview of the definition of the different subcells in a given simulation cell, in which the spheres denote the relevant metal atoms. The orange metal atoms are taken into account to define subcell 1, whereas the blue metal atoms are taken into account to define subcell 2. After a time t, it is possible that part of the total simulation cell volume is not accounted for by any of the different subcells.

Owing to the spatial disorder present in the material, the different atoms defining the simulation cell do not move cooperatively. As a result, it is not a priori guaranteed that the combination of the n_{subcells} subcells defined based on the procedure outlined above results in the total simulation cell. For instance, following this procedure, the two subcells in Supplementary Figure 2 move based on the positions of atoms 1 to 5. Given that the vector pointing from atom 1′ to atom 2′ no longer has to equal the vector pointing from atom 4′ to atom 5′ during the simulation, a mismatch between neighboring subcells may arise, as demonstrated with the gray area in Supplementary Figure 2. To quantify this possible mismatch, the missing volume V_{miss} is defined as

$$V_{\text{miss}} = \max_t \left\{ \left| V_{\text{tot}}(t) - \sum_{i=1}^{n_{\text{subcells}}} V_i(t) \right| \right\}. \quad (20)$$

In this definition, $V_{\text{tot}}(t)$ is the total volume of the simulation cell at time t, defined based on the cell vectors of this simulation cell, and $V_i(t)$ is the volume of subcell i at time t.

Furthermore, the normalized missing volume is defined as

$$V_{\text{miss}}^{\text{norm}} = \max_t \left\{ \frac{|V_{\text{tot}}(t) - \sum_{i=1}^{n_{\text{subcells}}} V_i(t)|}{V_{\text{tot}}(t)} \right\}. \quad (21)$$

In Table 2, the (normalized) missing volume is reported for all structures discussed in Figure 2 of the main text and the Supplementary Movies. For all considered structures, the normalized missing volume does not exceed 0.7%.

Supplementary Table 2: Missing volume and normalized missing volume for each of the $(N, P, \boldsymbol{\sigma}_{\text{a}} = \mathbf{0}, T)$ supercell simulations.

Material	Simulation cell	Thermodynamic conditions	V_{miss} [Å^3]	$V_{\text{miss}}^{\text{norm}}$ [%]
MIL-53(Al)	1×2×1	300 K, 40 MPa, empty host	0.0	0.000
MIL-53(Al)	2×2×2	300 K, 40 MPa, empty host	0.4	0.001
MIL-53(Al)	4×2×4	300 K, 40 MPa, empty host	108.7	0.056
MIL-53(Al)	6×2×6	300 K, 40 MPa, empty host	222.3	0.041
MIL-53(Al)	8×2×8	300 K, 40 MPa, empty host	154.0	0.021
DMOF-1(Zn)	8×2×8	300 K, 180 MPa, empty host	450.1	0.038
MIL-53(Al)-F	8×2×8	100 K, 80 MPa, empty host	2 533.6	0.218
MIL-53(Al)-F	8×2×8	300 K, 60 MPa, empty host	3 408.5	0.374
MIL-53(Al)-F	8×2×8	500 K, 60 MPa, empty host	3 979.4	0.436
CoBDP	8×2×8	300 K, 80 MPa, empty host	9 342.3	0.603
CoBDP	8×2×8	300 K, 80 MPa, 2 CH_4/unit cell	10 644.5	0.700
CoBDP	8×2×8	300 K, 80 MPa, 4 CH_4/unit cell	8 774.6	0.537

Supplementary Note 4 – Correlation between neighboring subcells in MIL-53(Al)

To study the size dependence of the phase coexistence observed in pillared-layered MOFs, only the two directions (*a* and *c*) that span the layers were systematically increased. In contrast, the third direction (*b*), corresponding to the 'pillar' direction, was kept constant as the 1D channels lay along this direction, resulting in subcells that behave very correlated along this direction during an lp-to-cp transition. This 1D correlated behavior was also observed, for instance, in the very recent work of Keupp et al. in which the thermal opening of DMOF-1(Zn) was studied for various crystallite sizes.[6]

To verify whether this statement also holds for MIL-53(Al), Supplementary Figure 3 illustrates the Pearson correlation coefficient between adjacent subcells (1–2), subcells that are separated by one intermediate subcell (1–3), subcells that are separated by two intermediate subcells (1–4), and subcells that are separated by three intermediate subcells (1–5). These correlation coefficients are extracted from a 250 ps $(N, P, \boldsymbol{\sigma}_{\mathrm{a}} = \mathbf{0}, T)$ simulation at $T = 300$ K and at a pressure P of either 0 MPa or 40 MPa, starting in the large-pore phase of MIL-53(Al). At 0 MPa, below the lp-to-cp transition pressure, the structure remains in its lp phase, whereas an lp-to-cp transition is observed for each simulation at 40 MPa.

Starting with the 4×2×4, 6×2×6, and 8×2×8 supercell simulations at 0 MPa, during which the MIL-53(Al) structure remains in the lp phase, one observes a relatively low correlation between neighboring subcells (1–2) along the *a* and *c* directions. This could be anticipated, as no large-amplitude structural reorganizations are observed during these simulations. For these simulations, the 1–2 correlation is the largest along the *b* direction, coinciding with the direction of the inorganic chains. The correlation between subcells furthermore tends to decrease the larger the distance between the considered subcells, as evidenced by the 1–3, 1–4, and 1–5 subcell correlations.

For the 2×2×2 supercell at 0 MPa, the correlation between neighboring cells is much larger, implying that the 2×2×2 supercell imposes a strong collective behavior on the dynamics of the MIL-53(Al) cell even when no phase transition is encountered during the simulation. This observation evidences that one needs to consider larger cells, such as the 4×2×4, 6×2×6, and 8×2×8 supercells, to allow the material to behave in an uncorrelated fashion. This was also observed in Figure 3 of the main text, for which the equations of state of the 2×2×2 supercell revealed a highly ordered MIL-53(Al) structure.

For the simulations at 40 MPa, depicted in the right-hand panes of Supplementary Figure

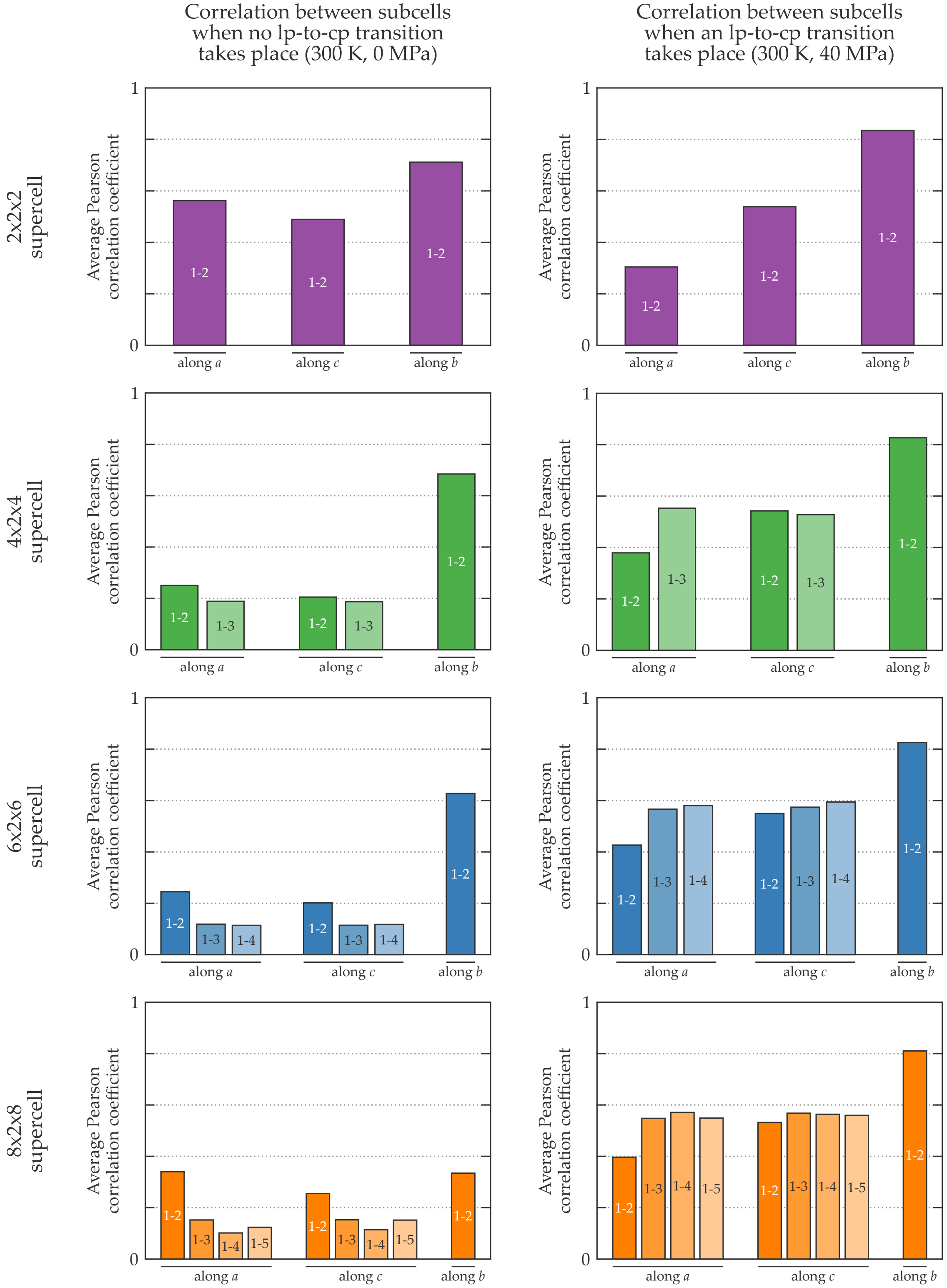

Supplementary Figure 3: Correlation between 1–n nearest neighboring subcells in different MIL-53(Al) supercells during a 250 ps $(N, P, \boldsymbol{\sigma}_{\mathrm{a}} = \mathbf{0}, T)$ simulation at $T = 300$ K. The panes on the left-hand side are collected at 0 MPa, below the lp-to-cp transition pressure, whereas the panes on the right-hand side are collected at 40 MPa, so that a transition takes place during the simulation. The inorganic chains lay along the b direction.

3, the correlations are systematically higher given that an lp-to-cp phase transition is induced, resulting in a large-amplitude structural reorganization with a given degree of collectivity. For all cell sizes, the correlation remains the highest for subcells along the inorganic chain, with a Pearson correlation coefficient that exceeds 80%, whereas the correlation for subcells along the in-layer directions remain below 60%. The high correlation between subcells along the b direction justifies our procedure not to further enlarge the simulation cell along this direction.

In contrast to the simulations at 0 MPa, the correlation between subcells at 40 MPa does not decrease the further these subcells are spaced apart. For instance, most 1–3 correlations at 40 MPa are larger than the corresponding 1–2 correlations. This can be attributed to the stronger correlation between diagonally adjacent subcells, as observed in Figure 2 of the main text. In contrast to 1–2 subcells, 1–3 separated subcells are connected by a common subcell along their diagonal. Given that the phase transition preferentially propagates along diagonals in the (a, c) plane given the winerack topology of the material, the 1–3 correlations are larger than the corresponding 1–2 correlations.

Supplementary Note 5 – Effect of the cell size on the MIL-53(Al) transition pressures

Given the substantial dependence of the pressure and free energy equations of state on the size of the MIL-53(Al) simulation cell, as reported in Figure 3 of the main text, one might be tempted to conclude that also the pressures necessary to induce a transition from the pure lp to the pure cp phase and vice versa are strongly affected by the simulation cell size. However, as reported in Supplementary Table 3, while increasing the simulation cell size does introduce metastable phase coexistence regions, the transition pressures extracted from the pressure equations of state are largely insensitive to the simulation cell size once at least a 2×2×2 simulation cell is considered.

The observed insensitivity of the lp-to-cp transition pressure on the simulation cell size is a result of simulating in the $(N, V, \boldsymbol{\sigma}_\mathrm{a} = \mathbf{0}, T)$ ensemble. It was demonstrated earlier that, when performing $(N, P, \boldsymbol{\sigma}_\mathrm{a} = \mathbf{0}, T)$ simulations instead, phase transitions could be induced in a 1×2×1 MIL-53(Al) nanocell even if the applied pressure P was well below the transition pressure.[7] These so-called premature phase transitions, a stochastic effect, are a direct consequence of the large fluctuations in the internal pressure during these $(N, P, \boldsymbol{\sigma}_\mathrm{a} = \mathbf{0}, T)$ simulations, which easily overcome the transition pressure. As a result, based on these $(N, P, \boldsymbol{\sigma}_\mathrm{a} = \mathbf{0}, T)$ simulations, one would substantially underestimate the lp-to-cp transition pressure. This effect is also observed in the left column of Supplementary Figure 4, which shows the volume of the different subcells and the volume of the total simulation cell normalized on the number of subcells at 300 K and at various pressures for a 1×2×1 MIL-53(Al) nanocell. Even at pressures as low as 0 MPa, the fluctuations in the instantaneous pressure induce the lp-to-cp phase transition. As the phase transition should only be induced at around 40–45 MPa, these $(N, P, \boldsymbol{\sigma}_\mathrm{a} = \mathbf{0}, T)$ simulations would lead to an underestimation of the lp-to-cp transition pressure by more than 40 MPa.

As the relative fluctuations in the instantaneous pressure are expected to decrease with in-

Supplementary Table 3: The 300 K lp-to-cp and cp-to-lp transition pressures for the different MIL-53(Al) simulation cells considered in Figure 3 of the main text.

Cell size	cp-to-lp transition pressure [MPa]	lp-to-cp transition pressure [MPa]
1×2×1	−238	55
2×2×2	−182	45
4×2×4	−180	44
6×2×6	−193	42
8×2×8	−179	43

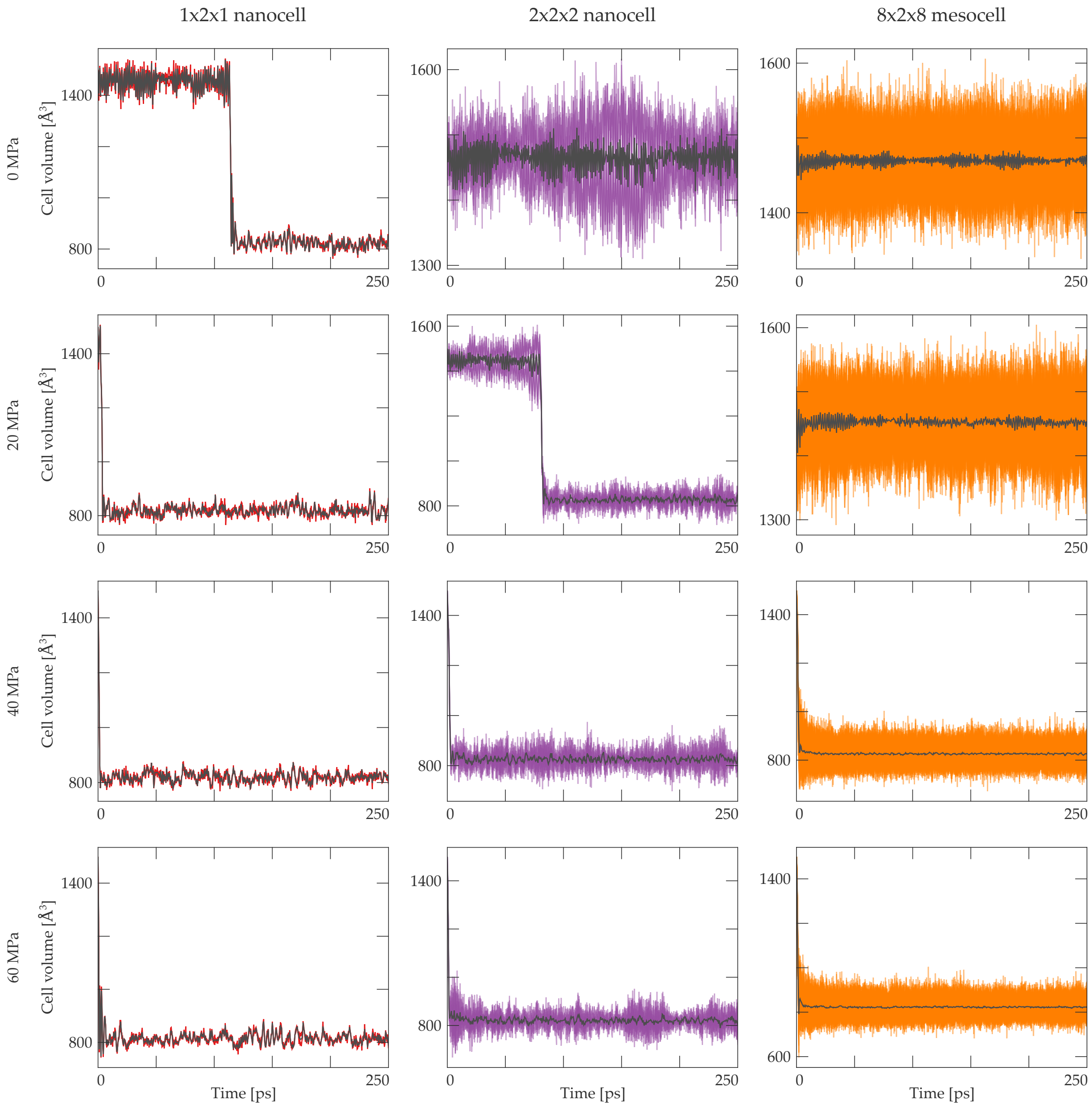

Supplementary Figure 4: Evolution of the volume of the different subcells (colored lines) and the volume of the total simulation cell normalized on the number of subcells (dark gray line) as a function of the simulation time during an $(N, P, \boldsymbol{\sigma}_\mathrm{a} = \mathbf{0}, T)$ simulation at 300 K for a 1×2×1 nanocell, a 2×2×2 nanocell, and a 8×2×8 mesocell of MIL-53(Al), starting in the large-pore phase. The pressures correspond, from top to bottom, to 0 MPa, 20 MPa (both below the transition pressure), 40 MPa (close to the transition pressure), and 60 MPa (above the transition pressure).

creasing cell size, also the probability of encountering these premature phase transitions should decrease. To verify this hypothesis, Supplementary Figure 4 also shows the volumes of the subcells and the normalized volume of the simulation cell for a $2\times2\times2$ nanocell and a $8\times2\times8$ mesocell of MIL-53(Al). For the $2\times2\times2$ nanocell, the 0 MPa simulation remains in the lp phase, while a premature phase transition is still induced at 20 MPa. For the $8\times2\times8$ mesocell, a phase transition is only induced at 40 MPa. As a result, if one were to extract the transition pressure directly from these $(N, P, \boldsymbol{\sigma}_\mathrm{a} = \mathbf{0}, T)$ simulations, the transition pressure would be size dependent, in contrast to the transition pressure extracted from our $(N, V, \boldsymbol{\sigma}_\mathrm{a} = \mathbf{0}, T)$ simulations.

As a final note, it should be observed that even for the $8\times2\times8$ mesocell, a phase transition is induced at 40 MPa, a pressure slightly below the transition pressure. This indicates that even for these large simulation cells the fluctuations in the pressure are not negligible. These remaining fluctuations complicate the direct simulation of the pressure quenching treatments proposed in the manuscript using $(N, P, \boldsymbol{\sigma}_\mathrm{a} = \mathbf{0}, T)$ simulations, an effect that is not present when using our pressure equations of state derived in the $(N, V, \boldsymbol{\sigma}_\mathrm{a} = \mathbf{0}, T)$ ensemble.

Supplementary Note 6 – Constructing the free enthalpy equations of state

As noted in the Methods section of the main text, all thermodynamic information can be extracted from the macroscopic pressure equation of state $P(N,T;V)$, which relates the volume V of a material with N atoms to the pressure P necessary to keep the material at this volume at a given temperature T.[7] The free energy $F(N,T;V)$ and free enthalpy $G(N,T,P;V)$ equations of state are derived from this pressure profile, according to

$$F(N,T;V) - F(N,T;V_{\text{ref}}) = -\int_{V_{\text{ref}}}^{V} P(N,T;V')\mathrm{d}V', \tag{22}$$

and

$$G(N,T,P;V) = F(N,T;V) + PV. \tag{23}$$

To predict the structure of a material under a given set of thermodynamic conditions, it is necessary to determine all stable, metastable, and transition states at these thermodynamic conditions. When considering a material under temperature and pressure control, the metastable states are found as the minima of the free enthalpy equation of state, whereas the transition states correspond to the maxima of this profile. The single stable state follows as that metastable state that has the lowest free enthalpy. This discussion can be readily extended to systems under temperature and volume control, for which the free energy is the relevant thermodynamic potential, as the free energy follows from the free enthalpy by requiring $P = 0$ MPa.

As the states of interest on the free enthalpy equation of state correspond to either minima or maxima on this profile, they can be found by requiring that

$$\begin{aligned} 0 &= \frac{\partial G(N,T,P;V)}{\partial V} &(24)\\ &= \frac{\partial F(N,T,P;V)}{\partial V} + P &(25)\\ &= -P(N,T;V) + P. &(26)\end{aligned}$$

Hence, the metastable and transition states of the free enthalpy equation of state are found at those volumes for which $P(N,T;V) = P$ or, in other words, at those volumes for which the pressure equation of state $P(N,T;V)$ coincides with the external pressure P (mechanical equilibrium). To distinguish between the metastable and transition states, it suffices to look at

the second-order derivative of the thermodynamic potential:

$$\frac{\partial^2 G(N,T,P;V)}{\partial V^2} = -\frac{\partial P(N,T;V)}{\partial V}. \tag{27}$$

As a result, the transition states, corresponding to maxima on the free enthalpy equation of state, are characterized by a positive slope in the pressure equation of state. Similarly, the metastable states are characterized by a negative slope in the pressure equation of state.

These equations immediately provide a very attractive way to determine the metastable and transition states of a given material by first determining the intersections between the pressure equation of state and a horizontal line at the external pressure and then investigating the slope of the equation of state at these intersections. To illustrate this, Supplementary Figure 5 shows

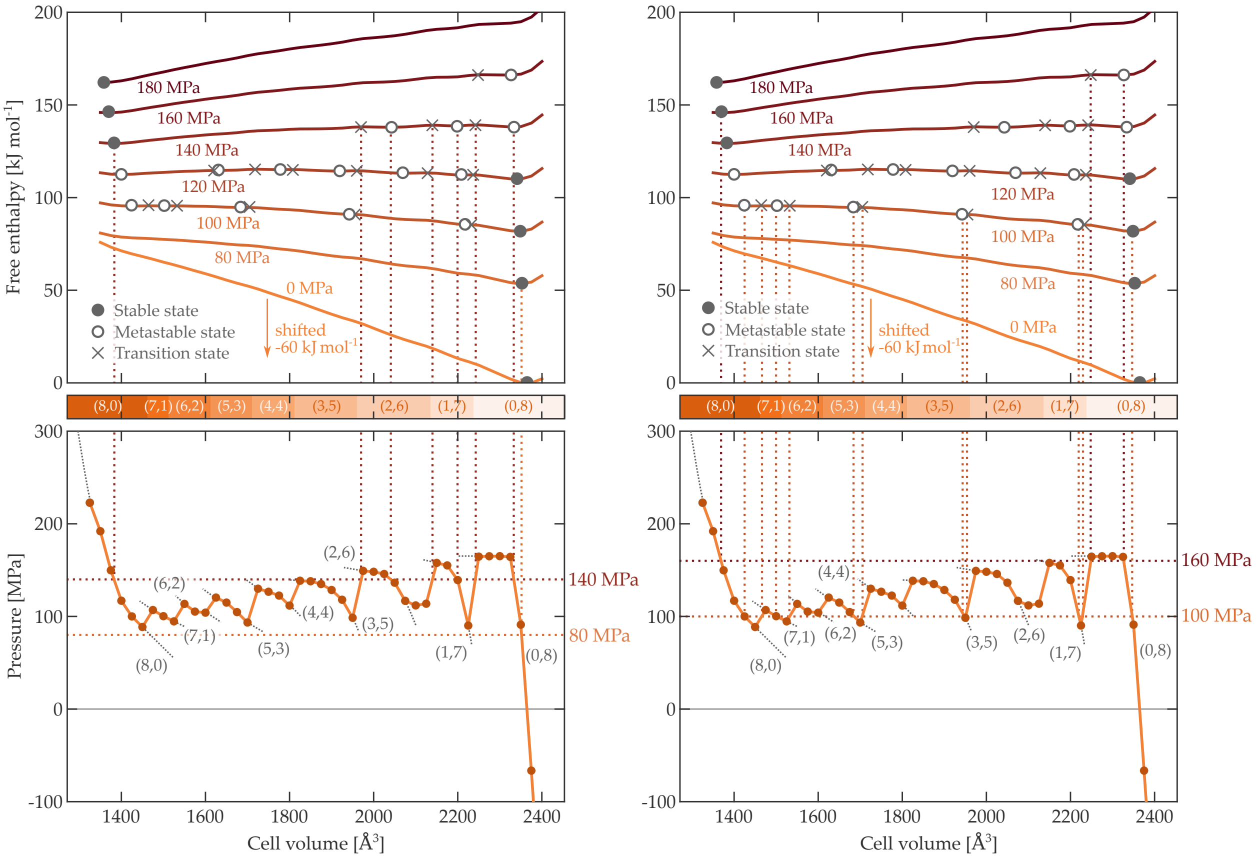

Supplementary Figure 5: Pressure (bottom) and free enthalpy (top) equations of state, with indication of the metastable (n_{cp},n_{lp}) phase coexistence regions as a function of the volume (middle) for an 8×2×8 mesocell of DMOF-1(Zn) at 300 K. The different (meta)stable states and transition states at different pressures are indicated on the free enthalpy equations of state with (open) circles and crosses, respectively. The 0 MPa free enthalpy profile is globally shifted over +60 kJ mol^{-1} to aid the visualization. The horizontal and vertical dotted lines are used to identify the (meta)stable and transition states, as explained in the text.

the pressure and free enthalpy equations of state for DMOF-1(Zn) at 300 K, which was also presented in Figure 4 of the main text. To determine the states of interest at 80 MPa, a horizontal line is drawn at this pressure in the bottom left pane, revealing only a single intersection with the pressure profile. As the slope of the profile is negative at this intersection, this point corresponds to a metastable state, which is also clear from the free enthalpy equation of state at this pressure. As this is the only metastable state at this temperature and pressure, it is necessarily the stable state at these conditions.

When increasing the pressure to 100 MPa, the same procedure can be followed, as outlined in the bottom right figure. At this pressure, the horizontal line intersects with the pressure profile at eleven volumes: five with a positive slope (transition states) and six with a negative slope (metastable states). From the six metastable states, the most stable state can be easily identified. This procedure also reveals the four (meta)stable and three transition states at 140 MPa, and the two (meta)stable and one transition state at 160 MPa.

Supplementary Note 7 – Convergence of the obtained equations of state

To verify whether the obtained equations of state are converged within the total simulation time of 1 ns (including a 100 ps equilibration), the convergence of these equations of state as a function of the simulation time is reported for the different materials discussed in the manuscript. Supplementary Figure 6 reports the convergence for different MIL-53(Al) simulation sizes, Supplementary Figure 7 reports the convergence for the DMOF-1(Zn) simulations, Supplementary Figure 8 reports the convergence for the MIL-53(Al)-F simulations at different temperatures, and Supplementary Figure 9 reports the convergence for the CoBDP simulations at different methane loadings. For all simulations, a fixed 100 ps equilibration and a production run with variable duration were considered. From these figures, one observes that convergence is systematically reached within the first 500 ps. As a result, the equations of state obtained after a production run of 700 ps and 900 ps are indistinguishable, confirming that the results reported here are well converged.

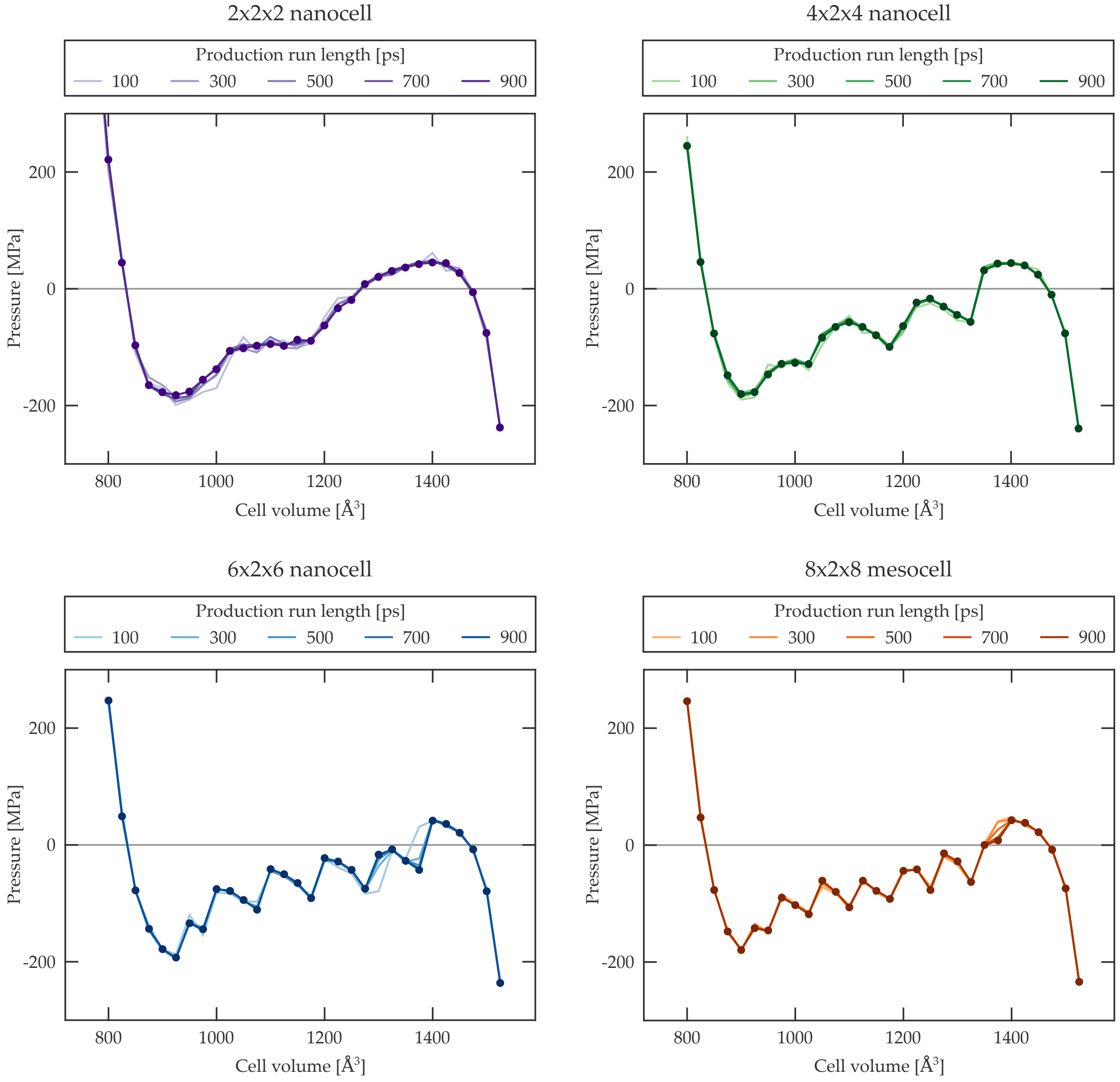

Supplementary Figure 6: Convergence of the pressure equations of state for the different MIL-53(Al) nanocells and mesoscells as obtained from a set of $(N, V, \boldsymbol{\sigma}_a = \mathbf{0}, T)$ simulations at 300 K, taking into account a fixed equilibration time of 100 ps followed by a production run with variable length.

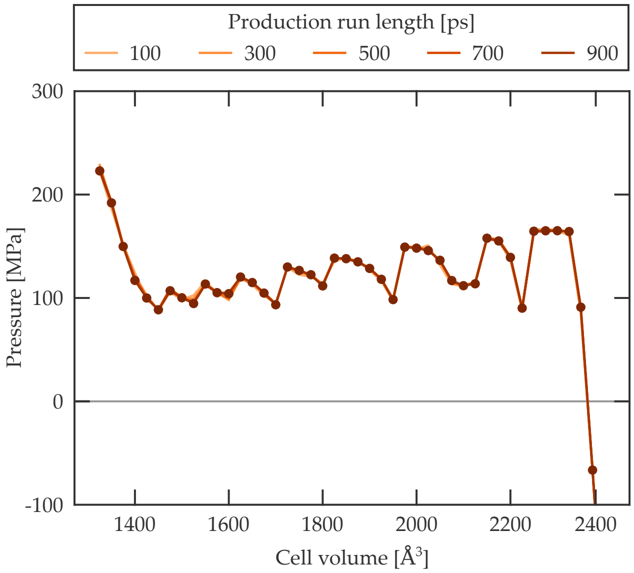

Supplementary Figure 7: Convergence of the pressure equation of state for the 8×2×8 DMOF-1(Zn) mesocell as obtained from a set of $(N, V, \boldsymbol{\sigma}_{\mathrm{a}} = \mathbf{0}, T)$ simulations at 300 K, taking into account a fixed equilibration time of 100 ps followed by a production run with variable length.

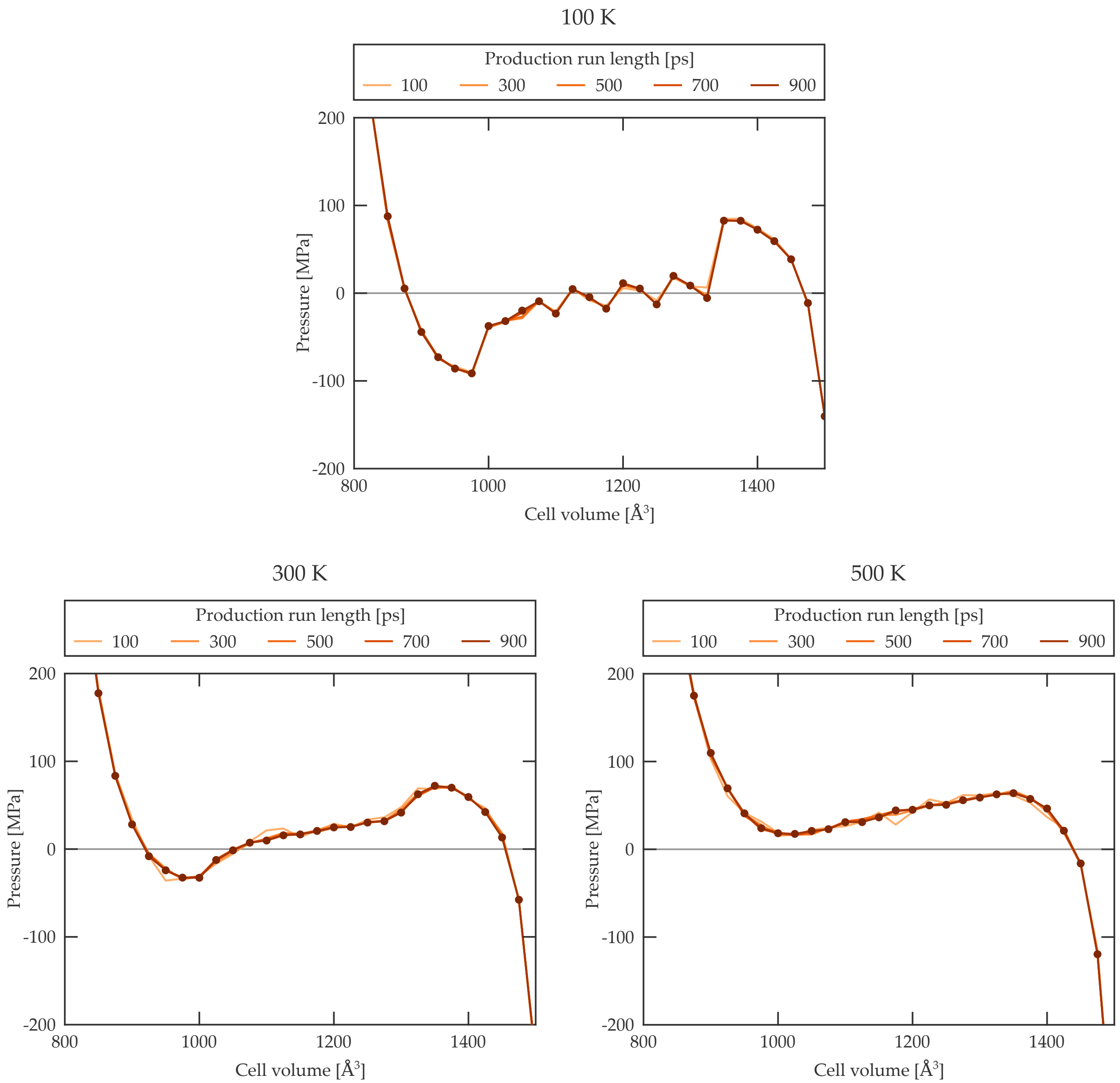

Supplementary Figure 8: Convergence of the pressure equations of state for the 8×2×8 MIL-53(Al)-F mesocell at different temperatures as obtained from a set of $(N, V, \boldsymbol{\sigma}_\text{a} = \mathbf{0}, T)$ simulations, taking into account a fixed equilibration time of 100 ps followed by a production run with variable length.

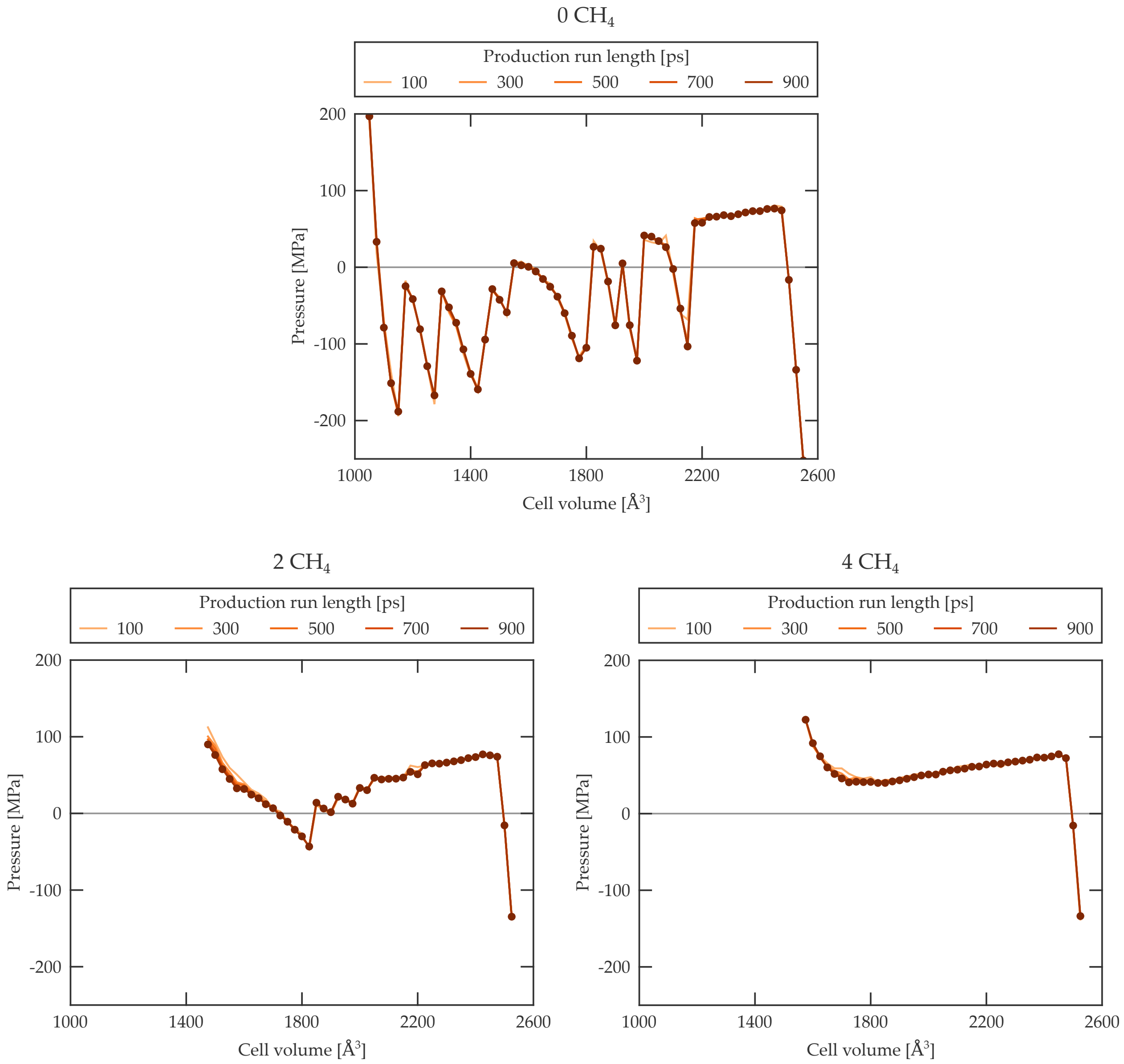

Supplementary Figure 9: Convergence of the pressure equations of state for the 8×2×8 CoBDP mesocell at different methane loadings as obtained from a set of $(N, V, \boldsymbol{\sigma}_\mathrm{a} = \mathbf{0}, T)$ simulations at 300 K, taking into account a fixed equilibration time of 100 ps followed by a production run with variable length.

Supplementary Note 8 – Robustness analysis of the obtained equations of state

As outlined in the Methods section, the pressure and free energy equations of state reported in the manuscript are determined from $(N, V, \boldsymbol{\sigma}_\mathrm{a} = \mathbf{0}, T)$ simulations that start from initial structures along a predefined volume grid. These initial structures are extracted from prior $(N, P, \boldsymbol{\sigma}_\mathrm{a} = \mathbf{0}, T)$ simulations. It is important, however, to verify that the here reported results are robust with respect to the choice of the volume grid, the initial $(N, P, \boldsymbol{\sigma}_\mathrm{a} = \mathbf{0}, T)$ simulations, and the choice of initial structures. In this Supplementary Note, this robustness will be demonstrated for MIL-53(Al) and CoBDP, the latter being chosen because of the additional degrees of freedom associated with the location of the guest molecules.

Robustness with respect to the volume grid and initial structures: MIL-53(Al)

To check the robustness with respect to the volume grid and initial structures of the pressure equation of state for MIL-53(Al), two independent $(N, P, \boldsymbol{\sigma}_\mathrm{a} = \mathbf{0}, T)$ simulations were performed to extract the initial structures. Afterwards, three sets of initial structures were extracted from these two simulations. A first set of initial structures was obtained by considering those structures from the first $(N, P, \boldsymbol{\sigma}_\mathrm{a} = \mathbf{0}, T)$ simulation that coincide best with a volume grid having a volume separation of 25 Å^3 (blue equations of state in Supplementary Figure 10). This first set of initial structures are also reported in Figure 3(d) of the manuscript. A second set of structures was obtained by considering the structures from the second and independent $(N, P, \boldsymbol{\sigma}_\mathrm{a} = \mathbf{0}, T)$ simulation that coincide best with a volume grid having a volume separation of 25 Å^3 (green equations of state in Supplementary Figure 10). Finally, a third set of initial structures was obtained by considering again the first $(N, P, \boldsymbol{\sigma}_\mathrm{a} = \mathbf{0}, T)$ simulation, but defining a denser volume grid with a separation of 10 Å^3 (orange equations of state in Supplementary Figure 10). As a result of this denser grid, the majority of the initial structures of this set are different from those of the first set.

From Supplementary Figure 10, it is clear that all initial structures yield very similar results. Where the different volume grids overlap, these results are in quantitative agreement, typically within a few MPa, whereas small deviations may result between the denser and the sparser volume grid given the larger interpolation necessary in the latter.

Furthermore, the results reported in Figure 3(d), which were also performed with the initial set of structures of set 1, are reproduced in Supplementary Figure 10 as the blue dotted line. For this dotted line, the exclusion rules for the noncovalent interactions were chosen differently,

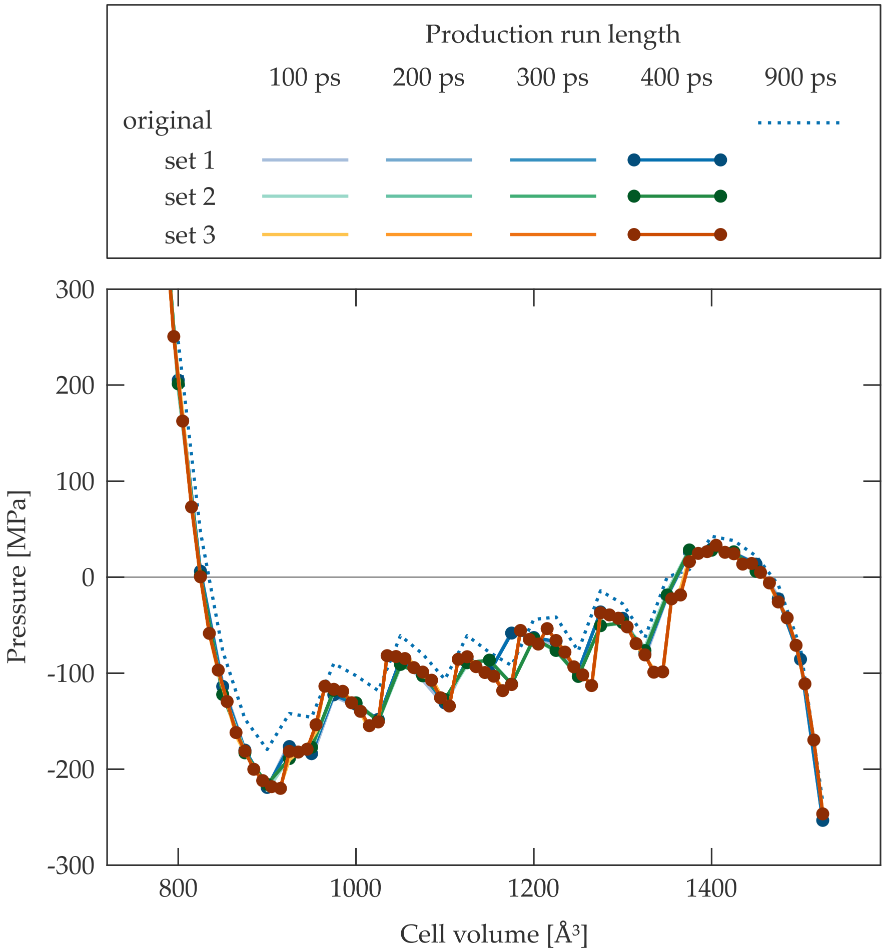

Supplementary Figure 10: Pressure equations of state for a 8×2×8 mesocell of MIL-53(Al) at 300 K obtained from 500 ps $(N, V, \boldsymbol{\sigma}_\mathrm{a} = \mathbf{0}, T)$ simulations using different volume grids and/or initial structures. The full lines represent the results obtained using three sets of initial structures that were chosen independently from each other. The blue and green structures are independently obtained along a volume grid with a volume spacing of 25 Å^3, whereas the orange structures are obtained along a volume grid with a reduced volume spacing of 10 Å^3. For these three sets of simulations, the results at intermediate simulation times (100 ps, 200 ps, 300 ps, and 400 ps) are shown using lighter shades to demonstrate convergence has been reached. The dotted blue line represents the original 1 ns results reported in Figure 3(d) of the manuscript, starting from the initial structures of set 1 but with a different description of the noncovalent interactions. For all simulations a 100 ps equilibration time has been taken into account.

effectively resulting in a slight alteration of the noncovalent interactions. As a result, comparing the two pressure equations of state for set 1 also allows one to quantify the sensitivity of our results on the description of the noncovalent terms. From Supplementary Figure 10, one observes that the difference between these two equations of state is not negligible near lower volumes, where the noncovalent interactions are more important. However, despite this, both equations of state predict the same qualitative phase coexistence behavior, confirming the robustness of our predictions.

Robustness with respect to the initial positions of the guest molecules: CoBDP

To confirm the robustness of the pressure equations of state with respect to the initial configurations of the methane molecules in CoBDP, four independent $(N, P, \boldsymbol{\sigma}_\text{a} = \mathbf{0}, T)$ simulations starting from different configurations were performed. Two of these simulations contained two methane molecule per conventional CoBDP unit cell, while the other two contained four methane molecules per conventional CoBDP unit cell. From these four simulations, four sets of initial structures for the $(N, V, \boldsymbol{\sigma}_\text{a} = \mathbf{0}, T)$ simulations were extracted, corresponding to two independent sets per guest loading.

The resulting pressure equations of state are reported as solid lines in Supplementary Figure 11. This figure demonstrates that very similar pressure equations of state are obtained for a given guest loading, independent of the initial structures, demonstrating the robustness of the method presented here. Supplementary Figure 11 furthermore also reports the initial pressure equations of state of Figure 6 in the manuscript, with different exclusion rules, as dotted lines. Also here the sensitivity with respect to the choice of initial structures is smaller than the sensitivity with respect to the noncovalent force field terms, similar to the earlier observations for MIL-53(Al).

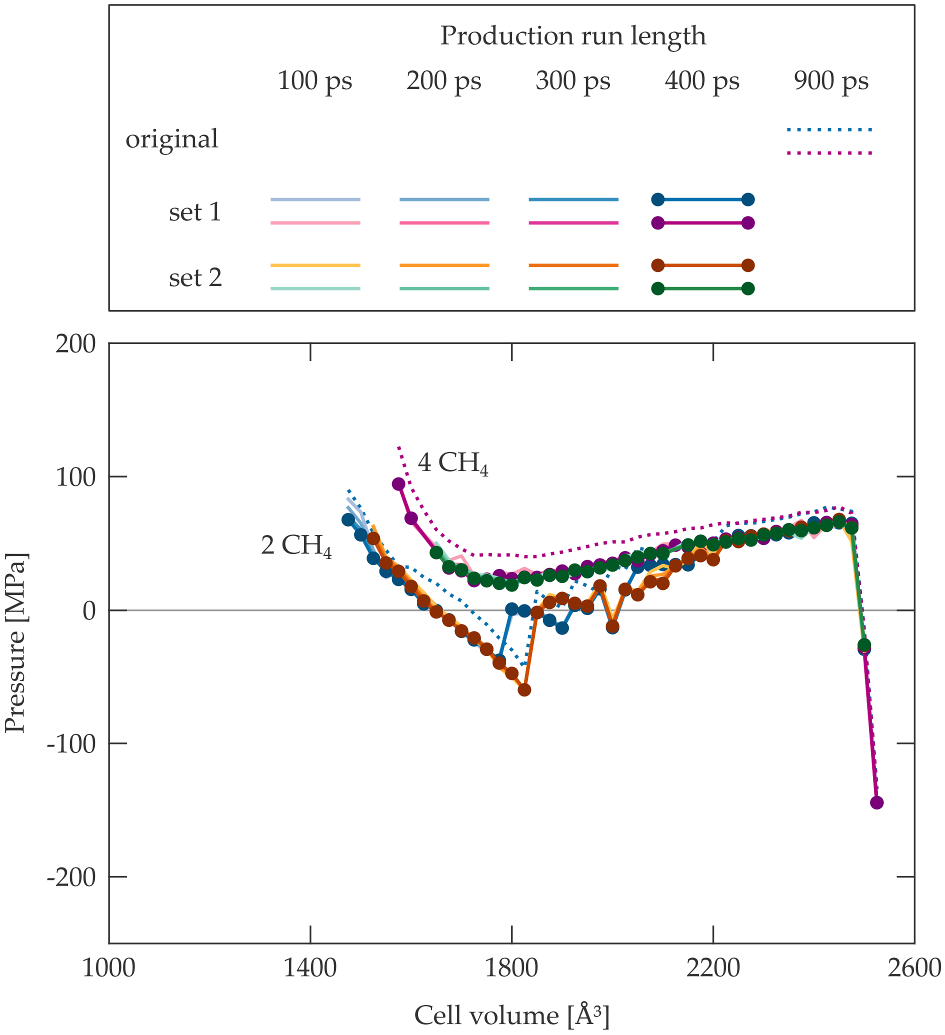

Supplementary Figure 11: Pressure equations of state for a 8×2×8 mesocell of CoBDP at 300 K and either two or four methane molecules per conventional unit cell, using 500 ps $(N, V, \boldsymbol{\sigma}_a = \mathbf{0}, T)$ simulations on two independent sets of initial structures. The dotted blue lines represent the original results reported in Figure 6 of the main manuscript. The full blue lines represent the results obtained using the same initial structures (set 1). The orange lines represent the structures obtained using a new set of initial structures, chosen independently from the original initial structures (set 2). For these simulations, results at intermediate simulation times (100 ps, 200 ps, 300 ps, and 400 ps) are shown using lighter shades to demonstrate convergence has been reached. The dotted lines represent the original 1 ns results reported in Figure 6 of the manuscript, starting from the initial structures of set 1 but with a different description of the noncovalent interactions. For all simulations a 100 ps equilibration time has been taken into account.

Supplementary Note 9 – Simulated X-ray diffraction patterns for the phase coexistence regions

To facilitate the experimental discovery of phase coexistence regions in soft porous crystals, X-ray diffraction patterns have been generated based on the simulated structures. To this end, a representative volume has been chosen for each material and each phase coexistence region and pure phase region. For each of these volumes, the average structure of the cell in the last 250 ps of the $(N, V, \boldsymbol{\sigma}_\mathrm{a} = \mathbf{0}, T)$ simulation at that volume has been extracted (262.5 ps for CoBDP). The crystallographic information files (CIFs) of the extracted structures are available at `https://github.com/SvenRogge/supporting-info`. Afterwards, the X-ray diffraction pattern for each of these average structures was determined using the freely available `genXRDPattern` program.[8] Here, the default copper Kα1 wavelength (1.54056 Å) was simulated, using a peak width of 0.572958 Å. The different X-ray diffraction patterns can be found in Supplementary Figures 12–14 for MIL-53(Al), in Supplementary Figure 15 for DMOF-1(Zn), in Supplementary Figure 16 for MIL-53(Al)-F at 100 K, and in Supplementary Figure 17 for the empty CoBDP structure.

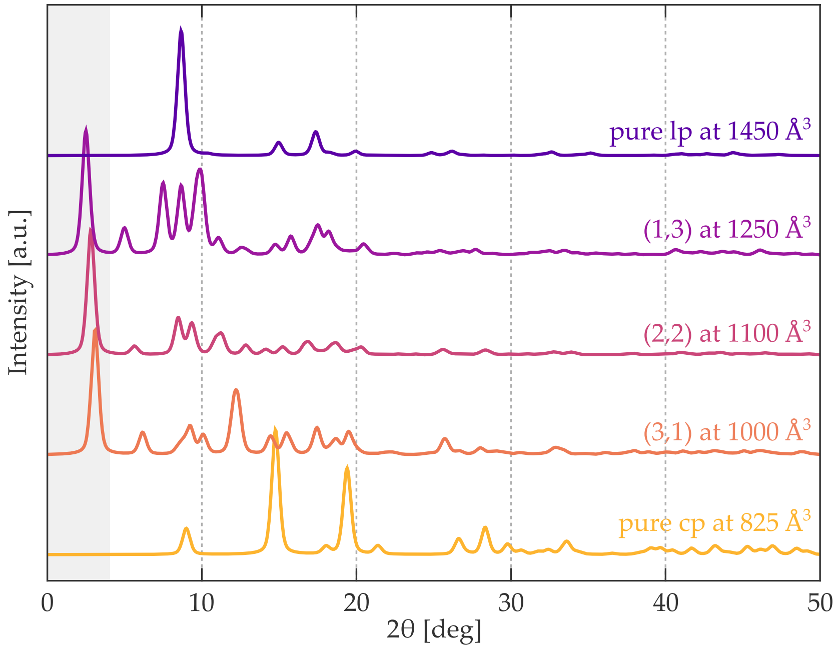

Supplementary Figure 12: Simulated X-ray diffraction patterns for the different pure phases and phase coexistence regions of a 4×2×4 cell of MIL-53(Al) at 300 K. The peaks in the grey area at low angles are a consequence of the periodic boundary conditions and would not be reproduced experimentally.

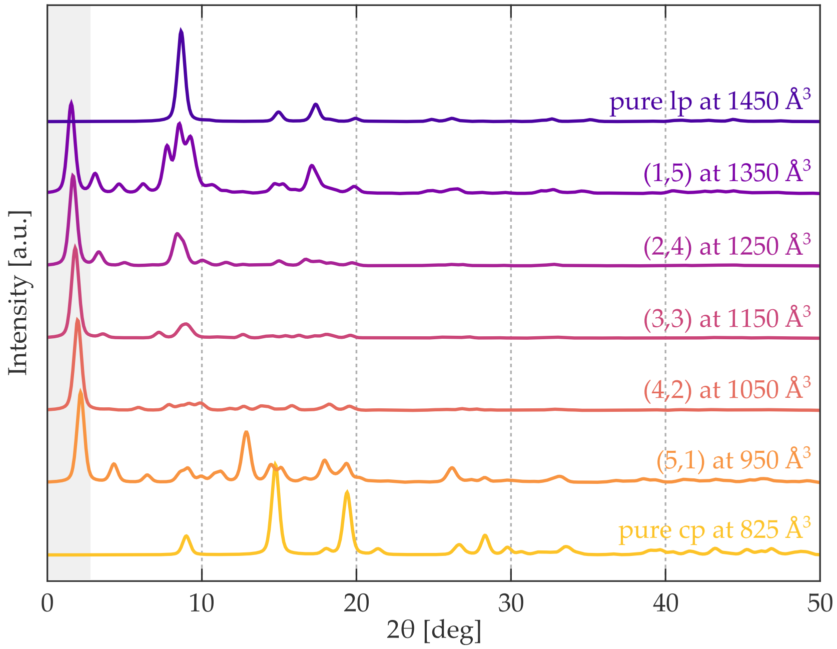

Supplementary Figure 13: Simulated X-ray diffraction patterns for the different pure phases and phase coexistence regions of a 6×2×6 cell of MIL-53(Al) at 300 K. The peaks in the grey area at low angles are a consequence of the periodic boundary conditions and would not be reproduced experimentally.

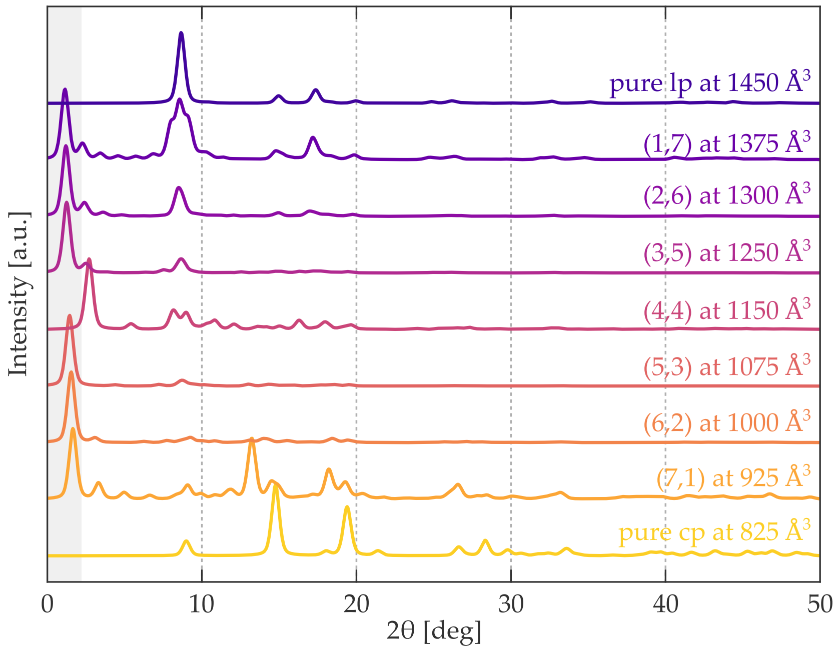

Supplementary Figure 14: Simulated X-ray diffraction patterns for the different pure phases and phase coexistence regions of a 8×2×8 cell of MIL-53(Al) at 300 K. The peaks in the grey area at low angles are a consequence of the periodic boundary conditions and would not be reproduced experimentally.

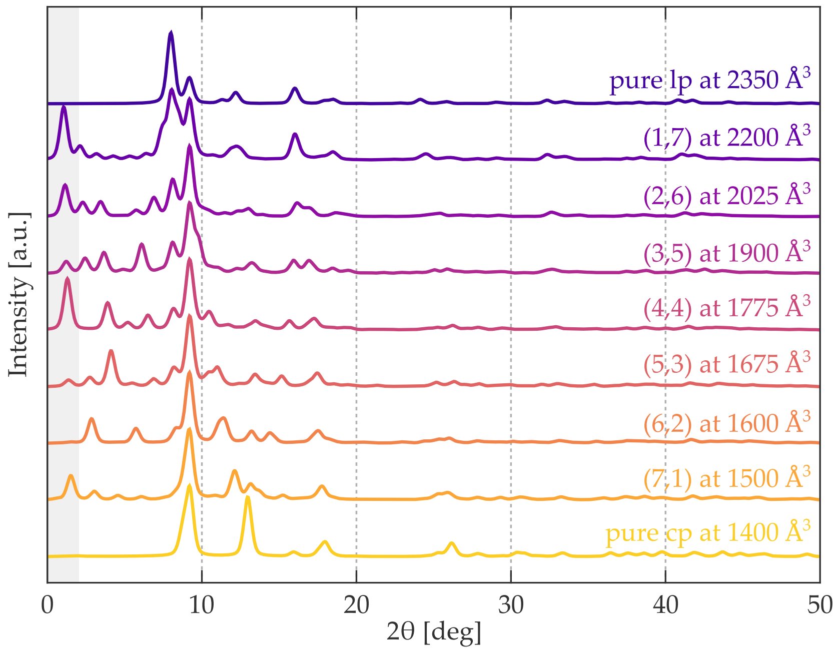

Supplementary Figure 15: Simulated X-ray diffraction patterns for the different pure phases and phase coexistence regions of a 8×2×8 cell of DMOF-1(Zn) at 300 K. The peaks in the grey area at low angles are a consequence of the periodic boundary conditions and would not be reproduced experimentally.

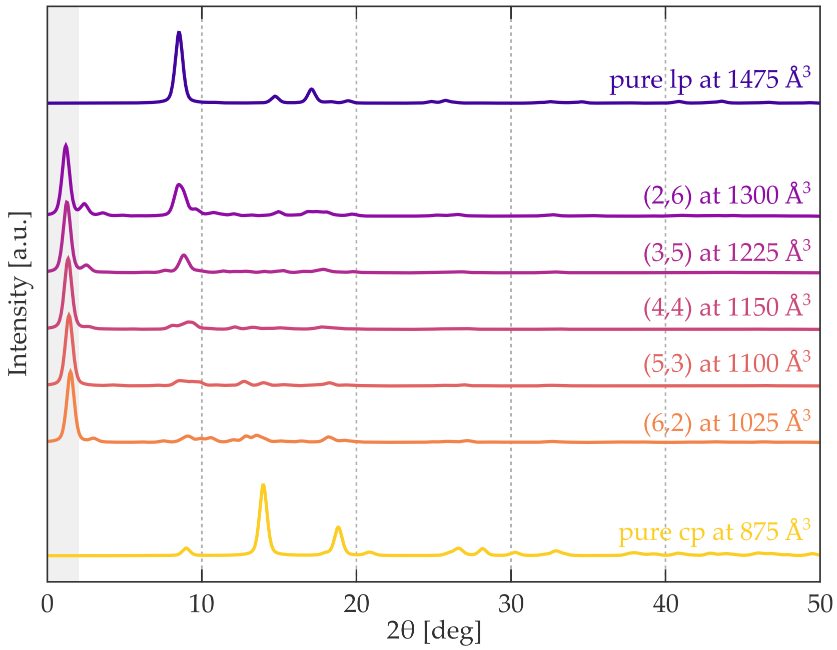

Supplementary Figure 16: Simulated X-ray diffraction patterns for the different pure phases and phase coexistence regions of a 8×2×8 cell of MIL-53(Al)-F at 100 K. The peaks in the grey area at low angles are a consequence of the periodic boundary conditions and would not be reproduced experimentally.

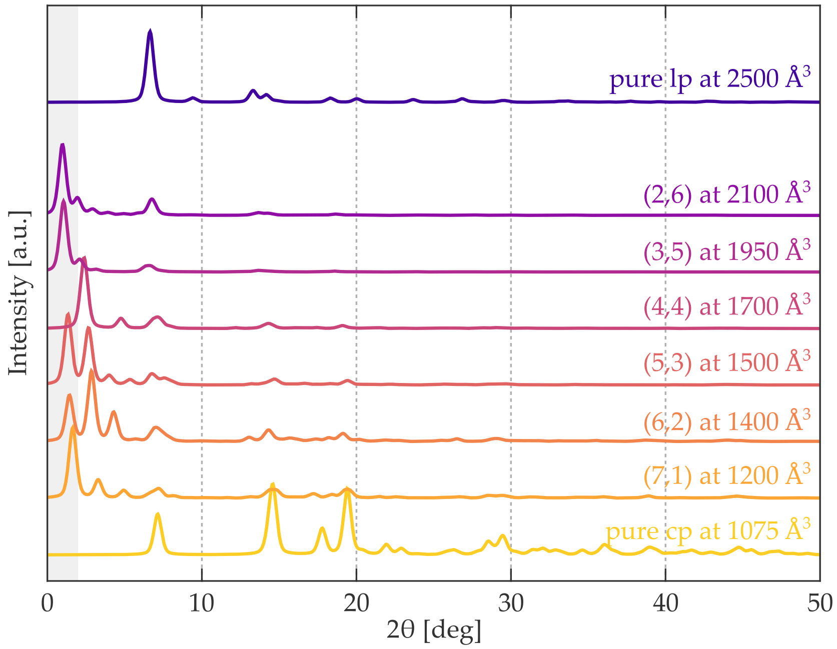

Supplementary Figure 17: Simulated X-ray diffraction patterns for the different pure phases and phase coexistence regions of an empty 8×2×8 cell of CoBDP at 300 K. The peaks in the grey area at low angles are a consequence of the periodic boundary conditions and would not be reproduced experimentally.

Supplementary References

(1) Wannapaiboon, S.; Schneemann, A.; Hante, I.; Tu, M.; Epp, K.; Semrau, A. L.; Sternemann, C.; Paulus, M.; Baxter, S. J.; Kieslich, G.; Fischer, R. A. Control of Structural Flexibility of Layered-Pillared Metal–Organic Frameworks Anchored at Surfaces. *Nat. Commun.* **10**, 346 (2019).

(2) Sakata, Y.; Furukawa, S.; Kondo, M.; Hirai, K.; Horike, N.; Takashima, Y.; Uehara, H.; Louvain, N.; Meilikhov, M.; Tsuruoka, T.; Isoda, S.; Kosaka, W.; Sakata, O.; Kitagawa, S. Shape-Memory Nanopores Induced in Coordination Frameworks by Crystal Downsizing. *Science* **339**, 193–196 (2013).

(3) Krause, S.; Bon, V.; Senkovska, I.; Többens, D. M.; Wallacher, D.; Pillai, R. S.; Maurin, G.; Kaskel, S. The Effect of Crystallite Size on Pressure Amplification in Switchable Porous Solids. *Nat. Commun.* **9**, 1573 (2018).

(4) Kundu, T.; Wahiduzzaman, M.; Shah, B. B.; Maurin, G.; Zhao, D. Solvent Induced Control Over Breathing Behavior in Flexible Metal-Organic Frameworks for Natural Gas Delivery. *Angew. Chem., Int. Ed.* **58**, 8073–8077 (2019).

(5) Miura, H.; Bon, V.; Senkovska, I.; Watanabe, S.; Ohba, M.; Kaskel, S. Tuning the Gate-Opening Pressure and Particle Size Distribution of the Switchable Metal–Organic Framework DUT-8(Ni) by Controlled Nucleation in a Micromixer. *Dalton Trans.* **46**, 14002–14011 (2017).

(6) Keupp, J.; Schmid, R. Molecular Dynamics Simulations of the "Breathing" Phase Transformation of MOF Nanocrystallites. *Adv. Theory Simul.* 1900117 (2019), DOI: 10.1002/adts.201900117.

(7) Rogge, S. M. J.; Vanduyfhuys, L.; Ghysels, A.; Waroquier, M.; Verstraelen, T.; Maurin, G.; Van Speybroeck, V. A Comparison of Barostats for the Mechanical Characterization of Metal–Organic Frameworks. *J. Chem. Theory Comput.* **11**, 5583–5597 (2015).

(8) Avery, P. *genXrdPattern*, Available online at http://github.com/psavery/genXrdPattern/.
