# Supplementary material for: Unraveling the thermodynamic criteria for size-dependent spontaneous phase separation in soft porous crystals
Source: Nat Commun. 2019 Oct 24;10:4842. doi: 10.1038/s41467-019-12754-w (PMC6813293; doi:10.1038/s41467-019-12754-w)
Supplement: Supplementary file 3 — Description of Additional Supplementary [file 41467_2019_12754_MOESM3_ESM.pdf]

Description of Additional Supplementary Files

File name: Supplementary Movie 1

Description: 5 ps (N, P, $\boldsymbol{\sigma}_{\mathrm{a}} = \mathbf{0}$, T) molecular dynamics simulation at 40 MPa and 300 K for a 1×2×1 nanocell of MIL-53(Al). The subcells are color coded according to the volume. The closed-pore phase (V < 900 Å^3), unstable region (900 Å^3 ≤ V < 1400 Å^3), and large-pore phase (V ≥ 1400 Å^3) are indicated by circles, crosses, and squares, respectively.

File name: Supplementary Movie 2

Description: 5 ps (N, P, $\boldsymbol{\sigma}_{\mathrm{a}} = \mathbf{0}$, T) molecular dynamics simulation at 40 MPa and 300 K for a 2×2×2 nanocell of MIL-53(Al). The subcells are color coded according to the volume. The closed-pore phase (V < 900 Å^3), unstable region (900 Å^3 ≤ V < 1400 Å^3), and large-pore phase (V ≥ 1400 Å^3) are indicated by circles, crosses, and squares, respectively.

File name: Supplementary Movie 3

Description: 5 ps (N, P, $\boldsymbol{\sigma}_{\mathrm{a}} = \mathbf{0}$, T) molecular dynamics simulation at 40 MPa and 300 K for a 4×2×4 nanocell of MIL-53(Al). The subcells are color coded according to the volume. The closed-pore phase (V < 900 Å^3), unstable region (900 Å^3 ≤ V < 1400 Å^3), and large-pore phase (V ≥ 1400 Å^3) are indicated by circles, crosses, and squares, respectively.

File name: Supplementary Movie 4

Description: 5 ps (N, P, $\boldsymbol{\sigma}_{\mathrm{a}} = \mathbf{0}$, T) molecular dynamics simulation at 40 MPa and 300 K for a 6×2×6 nanocell of MIL-53(Al). The subcells are color coded according to the volume. The closed-pore phase (V < 900 Å^3), unstable region (900 Å^3 ≤ V < 1400 Å^3), and large-pore phase (V ≥ 1400 Å^3) are indicated by circles, crosses, and squares, respectively.

File name: Supplementary Movie 5

Description: 5 ps (N, P, $\boldsymbol{\sigma}_{\mathrm{a}} = \mathbf{0}$, T) molecular dynamics simulation at 40 MPa and 300 K for a 8×2×8 mesocell of MIL-53(Al). The subcells are color coded according to the volume. The closed-pore phase (V < 900 Å^3), unstable region (900 Å^3 ≤ V < 1400 Å^3), and large-pore phase (V ≥ 1400 Å^3) are indicated by circles, crosses, and squares, respectively.

File name: Supplementary Movie 6

Description: 25 ps (N, P, $\boldsymbol{\sigma}_{\mathrm{a}} = \mathbf{0}$, T) molecular dynamics simulation at 180 MPa and 300 K for a 8×2×8 mesocell of DMOF-1(Zn). The subcells are color coded according to the volume. The closed-pore phase (V < 1450 Å^3), unstable region (1450 Å^3 ≤ V < 2300 Å^3), and large-pore phase (V ≥ 2300 Å^3) are indicated by circles, crosses, and squares, respectively.

File name: Supplementary Movie 7

Description: 5 ps (N, P, $\boldsymbol{\sigma}_a = \mathbf{0}$, T) molecular dynamics simulation at 80 MPa and 100 K for a 8×2×8 mesocell of MIL-53(Al)-F. The subcells are color coded according to the volume. The closed-pore phase (V < 1000 Å^3), unstable region (1000 Å^3 ≤ V < 1250 Å^3), and large-pore phase (V ≥ 1250 Å^3) are indicated by circles, crosses, and squares, respectively.

File name: Supplementary Movie 8

Description: 5 ps (N, P, $\boldsymbol{\sigma}_a = \mathbf{0}$, T) molecular dynamics simulation at 60 MPa and 300 K for a 8×2×8 mesocell of MIL-53(Al)-F. The subcells are color coded according to the volume. The closed-pore phase (V < 1000 Å^3), unstable region (1000 Å^3 ≤ V < 1250 Å^3), and large-pore phase (V ≥ 1250 Å^3) are indicated by circles, crosses, and squares, respectively.

File name: Supplementary Movie 9

Description: 5 ps (N, P, $\boldsymbol{\sigma}_a = \mathbf{0}$, T) molecular dynamics simulation at 60 MPa and 500 K for a 8×2×8 mesocell of MIL-53(Al)-F. The subcells are color coded according to the volume. The closed-pore phase (V < 1000 Å^3), unstable region (1000 Å^3 ≤ V < 1250 Å^3), and large-pore phase (V ≥ 1250 Å^3) are indicated by circles, crosses, and squares, respectively.

File name: Supplementary Movie 10

Description: 15 ps (N, P, $\boldsymbol{\sigma}_a = \mathbf{0}$, T) molecular dynamics simulation at 80 MPa and 300 K for an empty 8×2×8 mesocell of CoBDP. The subcells are color coded according to the volume. The closed-pore phase (V < 1200 Å^3), unstable region (1200 Å^3 ≤ V < 2350 Å^3), and largepore phase (V ≥ 2350 Å^3) are indicated by circles, crosses, and squares, respectively.

File name: Supplementary Movie 11

Description: 30 ps (N, P, $\boldsymbol{\sigma}_a = \mathbf{0}$, T) molecular dynamics simulation at 80 MPa and 300 K for a 8×2×8 mesocell of CoBDP with two methane molecules per unit cell. The subcells are color coded according to the volume. The closed-pore phase (V < 1200 Å^3), unstable region (1200Å^3 ≤ V < 2350 Å^3), and large-pore phase (V ≥ 2350 Å^3) are indicated by circles, crosses, and squares, respectively.

File name: Supplementary Movie 12

Description: 15 ps (N, P, $\boldsymbol{\sigma}_a = \mathbf{0}$, T) molecular dynamics simulation at 80 MPa and 300 K for a 8×2×8 mesocell of CoBDP with four methane molecules per unit cell. The subcells are color coded according to the volume. The closed-pore phase (V < 1200 Å^3), unstable region (1200 Å^3 ≤ V < 2350 Å^3), and large-pore phase (V ≥ 2350 Å^3) are indicated by circles, crosses, and squares, respectively.
